# A Portable, Cost-Effective and User-Friendly Instrument for Colorimetric Enzyme-Linked Immunosorbent Assay and Rapid Detection of Aflatoxin B_1_

**DOI:** 10.3390/foods10102483

**Published:** 2021-10-17

**Authors:** Wenzhi Tang, Yangchun Qi, Zhonghong Li

**Affiliations:** College of Food Science and Engineering, Northwest A&F University, Xianyang 712100, China; tangwenzhi@nwsuaf.edu.cn (W.T.); qiyc@nwafu.edu.cn (Y.Q.)

**Keywords:** ELISA, colorimetric, food safety, mycotoxin, rapid detection

## Abstract

Food analysis based on the enzyme-linked immunosorbent assay (ELISA) is simple, sensitive and rapid, but requires a costly colorimetric instrument. The aim of this work was to develop a portable, low-cost and user-friendly colorimetric instrument for colorimetric ELISA and aflatoxin B_1_ (AFB_1_) detection. The principle of the developed instrument was employing a light-emitting diode to generate the signal light and using a light-dependent resistor to measure the signal light absorbed by the oxidized 3,3′,5,5′-tetramethyl benzidine. The absorption spectra revealed that the solution absorbed signal light more strongly after reaction with H_2_SO_4_, and blue light would be favorably absorbed. Evaluations on the stability and accuracy of the instrument and interference from ambient light showed that the fabricated instrument was stable, accurate, capable of quantitative detection and insensitive to ambient light changes. In addition, this instrument is user-friendly since it could calculate and report the final amount of AFB_1_ to the operator. Measurements of maize and peanuts showed that the instrument provided as accurate results as the professional equipment. With the low fabrication cost (about RMB 129 or USD 20), portability, and user-friendliness, this instrument presents attractive potential in the rapid detection of AFB_1_.

## 1. Introduction

Foods are always at high risk of contamination by pathogens and their toxic secondary metabolites [1,2,3,4,5], posing severe threats to public health [6,7]. Although bactericidal treatments could effectively eliminate pathogens, the toxic metabolites would still remain in foods. A most toxic, widely distributed and well-known example is aflatoxin B_1_ (AFB_1_), a Group I carcinogen with the maximum limits of 0.5 to 20 μg kg^−^^1^ in different foods [4,8,9,10,11,12]. AFB_1_ is mainly produced by *Aspergillus* [11,13,14], and is thermally stable and would not be decomposed under normal cooking conditions [15]. Although many techniques, e.g., chromatography, mass spectrometry and electrochemical sensors, can accurately detect AFB_1_, most of them require sophisticated operations in clinical labs, costly instruments or professional personnel [16,17,18], limiting their applications in rapid detection. Therefore, it is necessary to make the quantitative detection of AFB_1_ more portable, low-cost and user-friendly [12,19].

The strategies based on specific recognition elements and subsequent conversion of recognition events into color changes present an attractive potential for rapid and low-cost detection of AFB_1_. One strategy is to use an antibody-based lateral flow test or aptamer-based lateral flow test, enabling the AFB_1_ detection by simply observing the test lines with naked eyes. However, the insufficient sensitivity and semi-quantitative results obtained by strip reader or image analysis software limit their application in AFB_1_ quantification [12,20]. Another strategy is the colorimetric enzyme-linked immunosorbent assay (ELISA), an analytical biochemistry assay using an immunological reaction for target recognition and an enzymatic reaction for signal generation and amplification [9,21,22,23,24]. Conventionally, a microplate reader is used to measure the colored solution at a specific wavelength and get the quantitative result. Although ELISA is considered to be simple and low-cost [25], the high cost of the instrument limits the application of ELISA in well-equipped laboratories by trained technical professionals [12,19], failing to meet the demand in remote or low-income areas. To meet the standards of quantitative, simple and low-cost detection for modern analytical systems [18,26,27], invertase, glucose oxidase and Pt have been used as the markers to modify the signal generation and amplification strategy of ELISA, thereby generating detectable signals for personal glucose meter [28], smartphone-based image processing [5] and pressure meter [29], respectively. These approaches are quantitative and low-cost, but require sophisticated modification of ELISA.

Light-emitting diodes (LEDs) and a photodiode, which could convert the intensity of light into an electrical signal, have been used in a colorimetric assay to detect organophosphorus pesticides [30]. Moreover, the light-dependent resistor (LDR) and LED are used to develop low-cost instruments that could analyze the colored sample solutions to detect ions in water and *staphylococcus aureus* in milk samples [31,32], respectively. The establishments of these methods are based on measuring the intensity of signal lights. As they are low-cost and do not require the modification of colorimetric assays, it would be attractive to investigate whether the LDR and LED are also feasible for the colorimetric analysis of ELISA readout and user-friendly quantification of AFB_1_ in food samples.

In this study, we developed a portable, low-cost and user-friendly instrument to analyze the colored solution of ELISA and detect AFB_1_. This instrument employed a blue LED and an LDR to measure the signal light absorbed by the yellow products generated in horseradish peroxidase (HRP)-catalyzed and H_2_O_2_-mediated oxidation of 3,3′,5,5′-tetramethyl benzidine (TMB), calculated and reported the AFB_1_ concentration to the operator. To verify its reliability, the instrument was used for the detection of AFB_1_ in peanuts and maize, and showed comparable performance as the commercial spectrophotometer.

## 2. Materials and Methods

### 2.1. Reagents and Equipment

HRP and TMB were purchased from Aladdin (Shanghai, China). The AFB_1_ ELISA kit was provided by Shenzhen Finder Biotech Co., Ltd. (Shenzhen, China). The kit was composed of 8-well ELISA plate strips containing immobilized AFB_1_, standard AFB_1_ solutions (0 to 0.48 μg L^−^^1^), a bottle of AFB_1_ antibody, a bottle of HRP-labeled secondary antibody, a bottle of washing buffer, a bottle of stop buffer and two bottles of substrates. Other reagents were of analytical grade and were used as received. The absorption spectra of colored solutions generated by HRP-catalyzed and H_2_O_2_-mediated oxidation of TMB were measured by a Shimadzu UV-2550 spectrophotometer. A Shimadzu UVmini-1240 spectrophotometer was used to measure the absorbance of sample solution at 450 nm.

### 2.2. Instrument Fabrication

**Figure 1:**
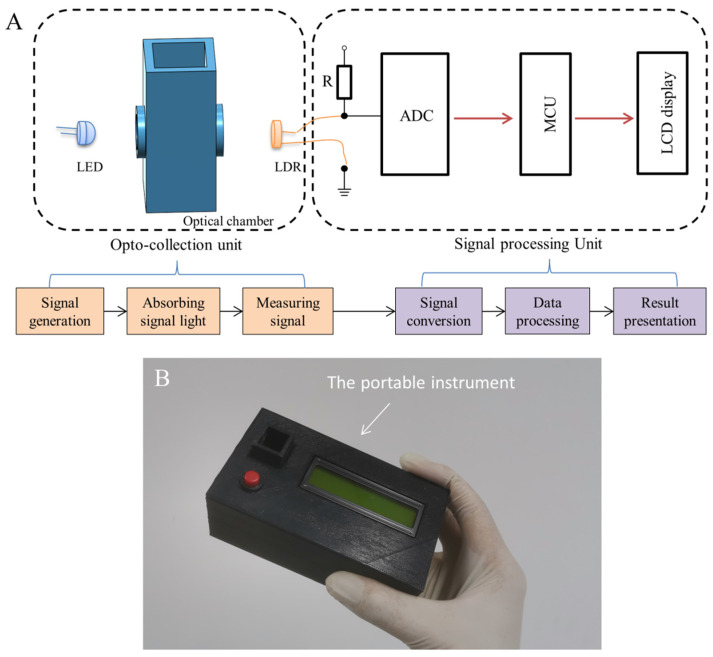
Design and fabrication of the instrument. (**A**) The structure of the optical chamber and schematic illustration of the portable instrument composed of the opto-collection unit (orange) and the signal processing unit (purple). (**B**) Photograph of the portable instrument.

As shown in Figure 1A, the portable instrument developed in this work was composed of two parts: an opto-collection unit to perform the optical measurement and generate electrical signals; a signal processing unit to process the generated signals, calculate AFB_1_ concentration and display results. The main body of the opto-collection unit was an optical chamber designed with Autodesk123D Design and made with polylactic acid via 3D printing technique. The signal light from the LED was absorbed by the sample placed in the optical chamber and then received by the LDR.

The signal processing unit was fabricated by using an ADS1115 16-Bit analog-to-digital converter (ADC), a microcontroller unit (MCU) using an ATmega328P microcontroller and an LCD1602 display. The ADC converted the LDR response into readable outputs for the MCU [31], and then the MCU calculated the change of optical density (OD) according to the Lambert–Beer law shown in Equation (1) [32]:

OD = lg (*I_0_/I*), (1)

where *I_0_* was the value for the first blank sample, and *I* was the value during the measurement. The LCD display was used to present the result. Moreover, a chargeable battery together with a power switch was used as the power source. Additionally, a 3D printed box (12.4 × 6.8 × 4.7 cm) was used as the instrument shell. Figure 1B was a photograph of the developed instrument. All components used in the fabrication are commercially available and the cost was about RMB 129 or USD 20 in total.

### 2.3. Characterization of the Instrument

The colored products generated by enzymatic oxidation of TMB were used to evaluate the performance of the instrument. To prepare the colored solution, 1 μL HRP (10 U mL^−^^1^, prepared in PBS) was added into 1 mL 0.1 mol L^−^^1^ PBS (pH 5.5) containing 100 mg TMB and 0.0315% H_2_O_2_. The solution was incubated for 10 min, and then acidified by 0.5 mL 1 mol L^−^^1^ H_2_SO_4_ to turn the blue solution into yellow. Cuvettes (10 mm, 50 μL) containing a series of diluted yellow solutions were measured by the portable instrument.

### 2.4. Standard Curve for AFB_1_ Detection

The standard curve for AFB_1_ was obtained according to the instruction of the AFB_1_ ELISA kit. Briefly, 50 μL of standard solution (0, 0.03, 0.06, 0.12, 0.24 and 0.48 μg L^−^^1^ AFB_1_), 50 μL of HRP-labeled secondary antibody solution and 50 μL of AFB_1_ antibody solution were added into the microwells of the ELISA plate strip, which were washed by using the washing buffer after an incubation of 30 min. Next, 50 μL of each substrate solution was added to initiate the catalytic reaction. After 15 min, 50 μL of stop buffer was added to terminate the enzymatic reaction. After that, the colored solution was transferred into a 50 μL cuvette with an optical path length of 1 cm to perform the measurement. The measured values were then normalized by Equation (2) provided in the instruction:

Normalized value = (A/A_0_) × 100%, (2)

where A was the value of a measured sample, and A_0_ was the value of the sample containing 0 μg L^−1^ AFB_1_. Then, the standard curve was obtained by using the normalized values.

### 2.5. Analysis of Food Samples

Six food samples (three Maize and three peanuts) were purchased from local markets (Yangling, China). According to the kit instruction, 2 g crushed sample was extracted with 5 mL of 70% methanol for 5 min, and centrifuged for 10 min at 4000 rpm (HC-3018 Centrifuge, ANHUI USTC Zonkia Scientific Instruments Co., Ltd., Hefei, China). After that, 0.5 mL extracted solution was diluted with 0.5 mL water. Then, the obtained sample solutions were analyzed by the ELISA kit. The resulted solutions were transferred into the cuvettes and measured by both the portable instrument and the UVmini-1240 spectrophotometer. The final amount of AFB_1_ in the sample was calculated by Equation (3):

Final amount = K × C,(3)

where K was the dilution factor and C was the value calculated using the measured absorbance and the standard curve. To facilitate the detection by the end-user, we further fitted the standard curve, Equations (2) and (3) into the equipment. In this way, the operator only needed to use a blank sample to subtract the background and a negative sample containing 0 μg L^−^^1^ AFB_1_ to provide a normalized value of 100%. Then, the instrument would directly report the AFB_1_ concentration in the sample when the sample was being measured. When a measured value was lower than the linear range of the standard curve, the readout of the instrument would be not detected.

## 3. Results and Discussion

### 3.1. Principle for the Detection of AFB_1_

**Figure 2:**
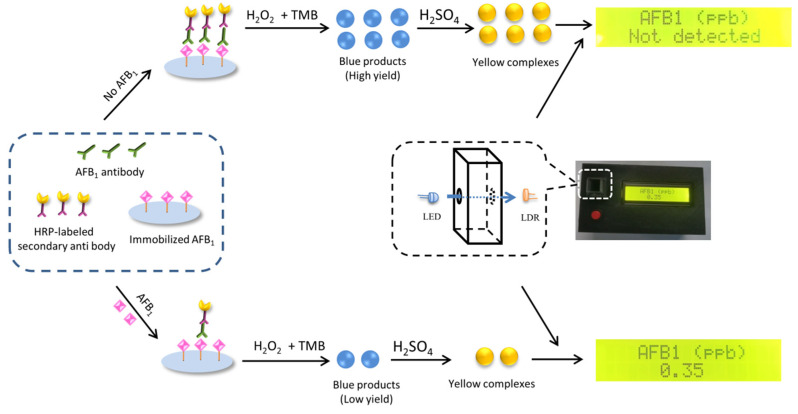
Schematic illustration of the principle for the AFB_1_ detection.

The detection was based on the colorimetric measurement of the colored readout of the AFB_1_ ELISA kit. As shown in Figure 2, in the absence of AFB_1_, the HRPs were bounded on the solid support as a result of the target recognition, thereby initiating catalyzed reaction of TMB to generate blue products. After adding H_2_SO_4_, the color of the products turned yellow. The yellow solution would absorb the signal light of the instrument. At the presence of AFB_1_, the competition between free and immobilized AFB_1_ resulted in less bounded HRPs, less yield of colored products and increased intensity of transmitted light. The instrument quantified the concentration of AFB_1_ according to the changes of transmitted light, and reported the final amount of AFB_1_ to the operator.

### 3.2. Instrument Evaluation

**Figure 3:**
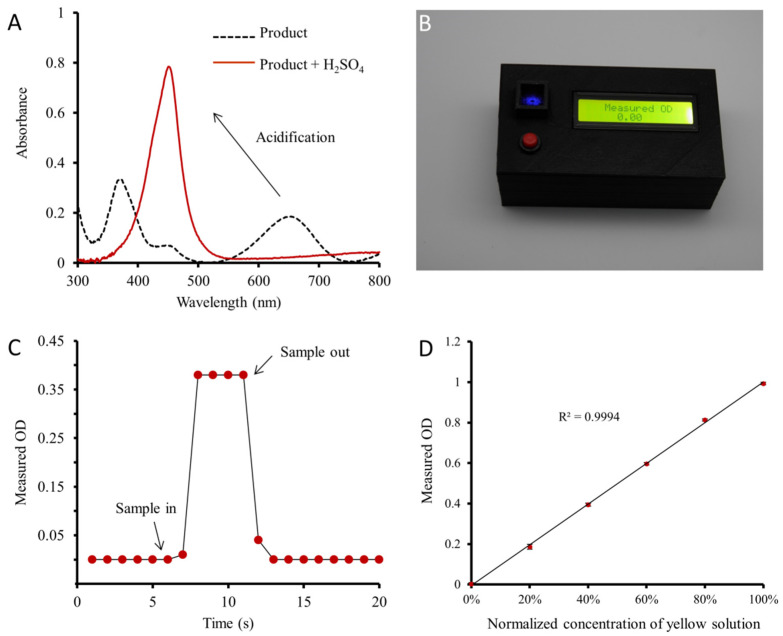
Evaluation of the portable instrument. (**A**) Absorption spectra of the colored compound before (dash line) and after (solid line) acidification. (**B**) A photo that a portable instrument using blue LED was measuring OD. (**C**) Response of the instrument during sample in/out. The sampling interval of the instrument was 1 s. (**D**) Measurement of a series of diluted sample solutions by the portable instrument (*n* = 3).

Because of the complicated instrumental design in generating a signal light with a specific wavelength, we firstly tested the feasibility to simplify the opto-collection unit by using an LED to provide the detection signal. To choose an optimal signal light, the absorption spectrum of the blue solution obtained by using HRP, H_2_O_2_ and TMB were measured. The blue solution showed a maximum absorption peak at ~650 nm (Figure 3A). After adding H_2_SO_4_, the color of the solution turned yellow, and the peak shifted to ~450 nm with an increased absorbance value. As this spectrum overlap with the wavelengths of blue lights, a blue LED was used as the light source of the instrument, obtaining a portable instrument measuring the OD of blue signal light (Figure 3B). A cuvette containing different solutions was used to evaluate the stability and accuracy of this portable instrument. As shown in Figure 3C and Video S1, the readout of the instrument was 0.00 and remained stable when the optical chamber was empty. Upon putting the cuvette containing sample solution into the optical chamber, the readout immediately reached 0.38 and remained at this value. When the cuvette was taken out of the chamber, the readout returned to 0.00 again, demonstrating a stable response of the portable instrument. Next, the accuracy of the instrument was investigated by measuring water, yellow sample solution and their mixtures containing 20%, 40%, 60% and 80% yellow solution, respectively. According to Figure 3D, the measured ODs were linearly proportional to the concentrations of the diluted solutions with a correlation coefficient of R^2^ = 0.9994, and all standard deviations (SDs) were less than 0.012 (*n* = 3). These results demonstrated that the absorption of unfiltered light from the blue LED also complied with the Lambert–Beer law, and the instrument was capable to provide a reliable and quantitative result for the colorimetric ELISA kit.

### 3.3. Effect of Ambient Light

**Figure 4:**
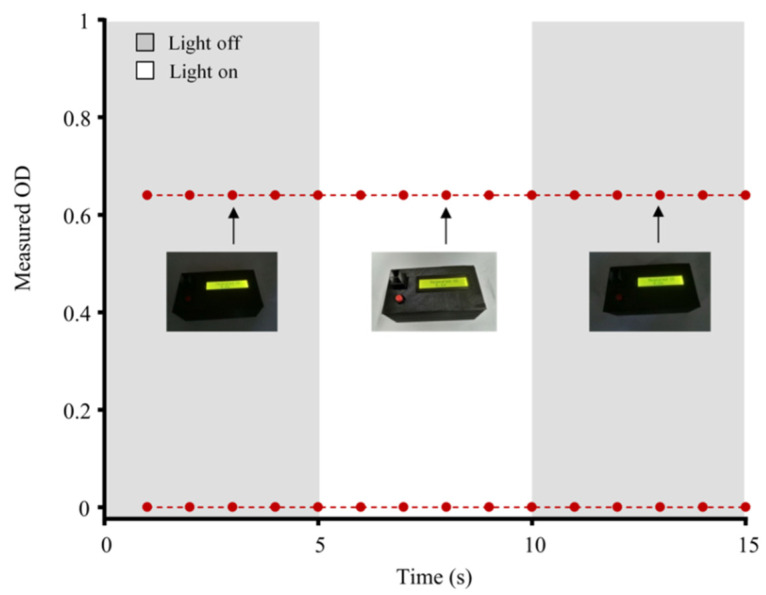
Effect of ambient light on the instrument. Insets were the photographs taken during the measurement.

The ambient light conditions may be changed during the measurement. As the opto-collection unit of this instrument was not placed in an enclosed environment, we investigated the effect of ambient light by measuring a blank solution and a colored solution, respectively. As shown in Figure 4, the light was turned on at 5 s and turned off at 10 s, but the responses of the instrument remained unchanged in both measurements, suggesting that the instrument was insensitive to the interference from ambient light changes. It indicated that there was no need to block ambient light for the opto-collection unit, simplifying the instrument design/fabrication and facilitating the operation, i.e., sample in/out processes.

### 3.4. Standard Curve for AFB_1_ Detection

**Figure 5:**
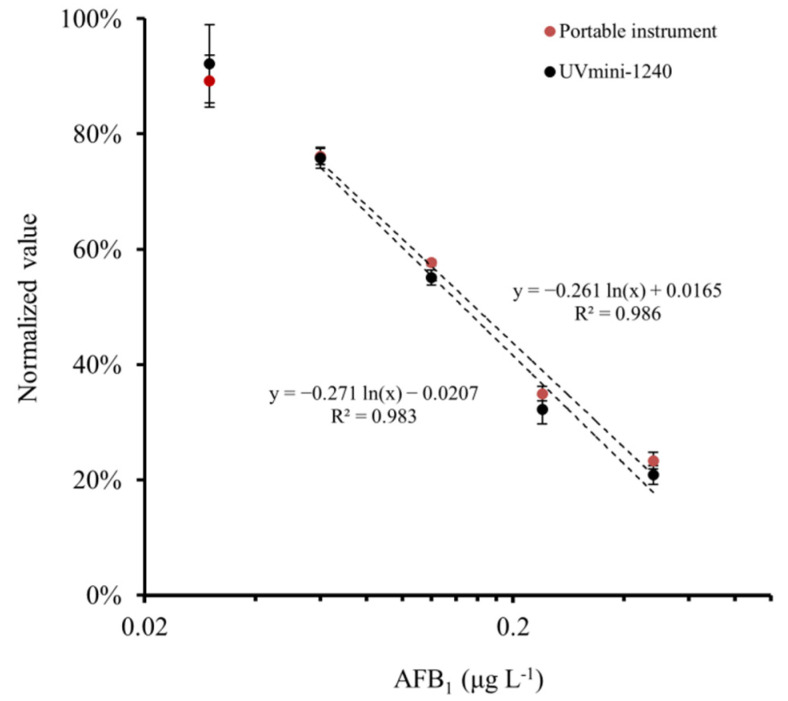
Calibration curve for AFB_1_ obtained by both the portable instrument and the professional spectrophotometer (*n* = 4).

To assess the applicability of the portable instrument for AFB_1_ detection, standard buffers containing 0, 0.03 0.06, 0.12, 0.24 and 0.48 μg L^−1^ AFB_1_ were analyzed by the AFB_1_ ELISA kit and measured by both the portable instrument and the UVmini-1240 spectrophotometer. As shown in Figure 5, the normalized values of the portable instrument decreased with the AFB_1_ concentration, and almost overlapped with the values calculated according to the absorbance from the UVmini-1240 spectrophotometer. Thus, the portable instrument was as accurate as the professional instrument.

Generally, this type of ELISA assay would give a sigmoid standard curve with a linear range between 20% and 80%. The values for the sample containing 0.03 μg L^−1^ AFB_1_ were out of this range and fluctuated significantly, which might not be reliable for quantitative detection. Hence, an empirical formula of y = −0.261 ln(x) + 0.0165 with the determine coefficient (R^2^) of 0.986 was established between 0.06 and 0.48 μg L^−1^ AFB_1_. The limit of detection (LOD) was determined to be 0.06 μg L^−1^, since its value was significantly different from that of 0.03 μg L^−1^ AFB_1_ (*p* < 0.05 by *t*-test). This LOD is capable to meet the requirement in monitoring AFB_1_ with the maximum limits in the range from 0.5 to 20 μg kg^−1^ [4,8,9,10,11,12].

### 3.5. Detection of AFB_1_ in Food Samples

**Figure 6:**
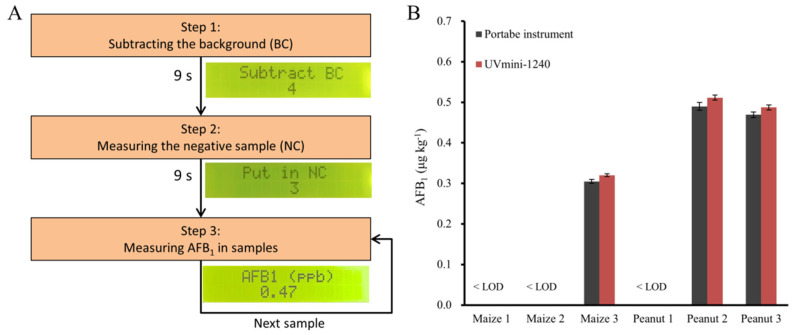
Rapid quantification of AFB_1_ in food samples. (**A**) Schematic illustration of the rapid quantification by using the portable instrument. Insets were the screenshots taken during steps 1, 2 and 3, respectively. (**B**) Detection of AFB_1_ in real samples (*n* = 3).

Given the complicated process to calculate the AFB_1_ concentration using the raw readout, we investigated the feasibility to automatically subtract the background, measure the ODs, normalize the measured values, calculate and output the amounts of AFB_1_ in the samples by fitting the standard curve, Equations (2) and (3) into the equipment, obtaining a protocol for the portable instrument (Figure 6A): (1) Subtracting the background (BC). An operator turns on the instrument, and puts a blank solution into the chamber during a 9 s countdown. (2) Measuring the negative control (NC) containing 0 μg kg^−1^ AFB_1_. The operator takes out the blank sample and then puts in the NC, which is requested to be accomplished in a 9 s countdown as well. (3) Measuring the sample. The operator takes out the NC from the optical chamber, puts the sample into the chamber, and then reads the AFB_1_ concentration from the LCD display. For the detection of more samples, the operator only needs to place them into the chamber one by one. Because of this proposed protocol, the instrument could be easily calibrated by using a blank solution and a negative sample containing 0 μg L^−^^1^ AFB_1_, and could report the AFB_1_ concentration without additional data management by operators.

Peanuts and maize were used to evaluate the feasibility of this user-friendly strategy. The instrument readouts were compared with the results calculated by using the absorbance values from the spectrophotometer. As shown in Figure 6B, the readouts of the portable instrument were similar to the results obtained by the spectrophotometer, demonstrating the capability of the developed instrument for rapid quantification of AFB_1_ in food samples. Compared with the costly instrument operated by the trained personnel in an advanced laboratory, this portable instrument provides a low-cost and user-friendly alternative to facilitate the quantitative detection of AFB_1_. On the other hand, ELISA tests are generally performed in a high-throughput manner by using the microplate reader, but this developed instrument has to measure the samples one by one. In this case, further improvement can be focused on the realization of rapid and multi-channel measurements to enhance detection efficiency.

## 4. Conclusions

In this study, a portable, cost-effective and user-friendly instrument was fabricated for rapid quantification of AFB_1_. The opto-collection unit made by using an LDR and blue LED obeyed the Lambert–Beer law, and the signal processing unit was able to accurately convert the response of the LDR sensor into quantitative readouts in a user-friendly way. Integration of the two units simplified the instrumental design, reduced the manufacturing cost and provided a portable and easy-to-use colorimetric instrument. With the low manufacturing cost (about RMB 129 or USD 20) and the user-friendly protocol of the instrument, users could rapidly and easily quantify the AFB_1_ in food samples. Additionally, the application of this instrument would be extended to various targets by using different ELISA kits, presenting considerable promise towards quantitative and cost-effective detection of contaminants in foods.

## Data Availability

Data are contained within the article and Supplementary Material.

## Supplementary Materials

The following are available online at https://www.mdpi.com/article/10.3390/foods10102483/s1, Video S1: Response of the portable instrument during the measurement.
